# Risk Factors Associated with Neonatal Sepsis: A Case Study at a Specialist Hospital in Ghana

**DOI:** 10.1155/2019/9369051

**Published:** 2019-01-01

**Authors:** Peter Adatara, Agani Afaya, Solomon Mohammed Salia, Richard Adongo Afaya, Kennedy Diema Konlan, Eric Agyabeng-Fandoh, Ethel Agbinku, Esther Aku Ayandayo, Irene Gifty Boahene

**Affiliations:** ^1^School of Nursing and Midwifery, University of Health and Allied Sciences, Ho, Ghana; ^2^Department of Nursing, Kwame Nkrumah University of Science and Technology, Kumasi, Ghana

## Abstract

Worldwide, neonatal sepsis accounts for an estimated 26% of under-five deaths, with sub-Saharan Africa having the highest mortality rates. Though worldwide neonatal deaths have decreased by over 3.6 million per year since 2000, neonatal sepsis remains a notable hindrance to the progress in the decline of cause-specific mortality rates especially in sub-Saharan Africa. This study aimed at examining the risk factors of neonatal sepsis at the Trauma and Specialist Hospital, Winneba. The study was an unmatched case control retrospective study. Cases were neonates who had sepsis with their index mothers and controls were neonates who did not have sepsis with their index mothers. Neonatal and maternal medical records were retrieved from January to December 2017. Data abstraction lasted for one month and 2650 folders for the neonates and their index mothers were retrieved. Nine hundred (900) neonatal folders were considered valid for the study and likewise for the maternal folders. One hundred and three (103) folders were considered cases while 797 were considered as controls. Data were entered using the Statistical Package for Social Sciences Version 22. Logistic regression was used to determine the risk of neonatal sepsis. Maternal factors that predicted the occurrence of sepsis among neonates were parity (*p*<0.027), mode of delivery (*p*<0.001), bleeding disorder (*p*<0.001), and PROM (*p*<0.001). Neonatal risk factors which predicted the occurrence of sepsis were APGAR score in the first and fifth minute (*p*<0.001), resuscitation at birth (*p*<0.004), duration of stay in the facility (*p*<0.001), and neonatal age on admission (*p*<0.001). The study found both maternal and neonatal factors to have a strong association with the risk of developing neonatal sepsis. Encouraging maternal antenatal care utilization would help identify the risk factors during prenatal and postnatal care and appropriate interventions implemented to reduce the likelihood of the neonate developing sepsis.

## 1. Introduction 

Worldwide, neonatal sepsis accounts for an estimated 26% of under-five deaths, with sub-Saharan Africa (SSA) having the highest mortality rates. Sub-Saharan Africa has an uneven burden of neonatal mortality, leading to an estimated 49.6% of all under-five deaths in 2013 [1]. The third Sustainable Development Goal for child health aims to end preventable deaths of newborns and children under five years of age by 2030; this goal may not be attained without significant reduction of neonatal mortalities directly related to infection in developing countries [2].

Globally, neonatal mortalities have declined since 2000 by over 3.6 million per year probably due to the declines in the incidence of major disease conditions (pneumonia and diarrhea). Despite the decrease in neonatal deaths, research shows that neonatal sepsis remains a remarkable hindrance to the progress in the decline of cause-specific mortality rates in the world, particularly Africa [1]. Previous reports indicate a range of 380,000–2,000,000 yearly cases of neonatal sepsis in SSA and annual related neonatal deaths of 270,000 [3–5]. This highlights the substantial burden of neonatal sepsis in the world, particularly SSA. However, an estimation of the exact burden of neonatal sepsis in SSA is limited by uncertainty in the diagnosis and estimations of the incidence. Despite the heavy burden of neonatal deaths related to neonatal sepsis, research shows that neonatal sepsis receives less substantial international investment as a public health priority compared with other major conditions [2].

Neonatal sepsis arises when pathogenic microorganisms gain entry into the bloodstream causing devastating systemic infection within the first 28 days of life [3, 6]. It is observed that birth asphyxia, prematurity, low birth weight, and other factors such as delivery settings, type of delivery, antenatal care received, newborn mixed feeding, and some cultural practices for cord care are believed to contribute to the incidence of neonatal sepsis [7–9] across the world causing morbidity and mortality among neonates. Several maternal and neonatal risk factors have been related to neonatal sepsis. In Ghana, statistics show that there was an estimated neonatal mortality rate of 28 per 1,000 live births in 2014 [10] which further declined to 26.9 per 1000 live births in 2016. Although infant and under-5 mortality rates in Ghana have been declining steadily since the early 2000s, neonatal mortality rates have increased [10]. Newborn deaths contributed to about 46% of under-5 deaths in the country in 2014. Current report shows that infection (31%) is the leading cause of neonatal mortality followed by prematurity (29%) and complications during birth (27%), all contributing to about 87% of neonatal mortality in the country. Newborn mortality rate is relatively high among the regions in Ghana and the Central Region of Ghana has one of the highest mortality rates of about 36 deaths per 1000 live births [10].

Although previous studies have looked at the common causative agents of neonatal sepsis with their sensitivity patterns, there are limited studies on the risk factors of neonatal sepsis in Ghana, particularly in the study setting. Early identification of these risk factors of neonatal sepsis and early institutional interventions can reduce neonatal mortality and morbidity rates in the country and the world at large. This study aimed at assessing the risk factors of neonatal sepsis in the Trauma and Specialist Hospital, Winneba, Ghana.

## 2. Materials and Methods

### 2.1. Study Design

This was an unmatched retrospective case control study conducted among 900 neonates born within January to December 2017 at the Trauma and Specialist Hospital, Winneba, Ghana. Neonatal sepsis (cases) were diagnosed based on hematological criteria (total leukocyte count, neutrophil count, erythrocyte sedimentation rate (ESR), and platelet count) and Integrated Management of Neonatal and Childhood Illness (IMNCI) clinical features (of either fever (≥37.5°C) or hypothermia (≤35.5°C), fast breathing (≥60 breathes per minute), not feeding well, severe chest in-drawing, movement only when stimulated, convulsion, lethargy, or unconsciousness). Some diagnoses were made based on the clinical features due to parents not being able to afford laboratory services.

### 2.2. Study Setting

The setting of the study was the Trauma and Specialist Hospital, Winneba. The Trauma and Specialist Hospital serves as the regional hospital for the Central Region of Ghana. The pediatric ward doubles as the Neonatal Intensive Care Unit (NICU) with 4 incubators and 5 phototherapy machines. The NICU has two cubicles/ward designated for only neonatal cases, 5 beds, and 4 cots.

### 2.3. Data Abstraction

Data abstraction was done by 3 of the current study authors and 4 trained research assistants. The neonatal and maternal medical records were retrieved from the Records Department (RD). Each day about 100 folders (medical records) of the neonates with their index mothers were taken from the RD. Folders that were retrieved without either the neonatal or maternal index folders were exempted from the study. Folders with the index maternal or neonatal information were considered for data abstraction. Abstracted data were cross-checked by authors to make sure the required information was in the tool. Data abstraction lasted for one month and 2650 folders for the neonates and their index mothers were retrieved; 900 neonatal folders were considered valid for the study and likewise for the maternal folders. One hundred and three (103) folders were retrieved and considered cases while 797 folders were considered as controls.

### 2.4. Data Abstraction Tool

A semistructured data abstraction tool was developed based on reviewed related literature on risk factors of neonatal sepsis [11, 12]. The data abstraction tool was composed of two parts: part one consisted of neonatal characteristics and part two consisted of maternal characteristics. Neonatal characteristics comprised neonatal age on admission, sex, gestational age, birth weight, APGAR score at the first and fifth minute, crying immediately at birth, resuscitation at birth, and duration of stay on admission. Maternal characteristics included age of mother, marital status, educational level, occupational status, parity, mode of delivery, number of antenatal visits, hypertensive disorders, bleeding disorder, UTI/STI, and PROM.

### 2.5. Data Analysis

Data were entered into the IBM Statistical Package for Social Sciences Version 22 (SPSS ver. 22) for analysis. To determine the relations between independent variables and dependent variables, Pearson's chi-squared test was used for analysis. Also in determining the risk of neonatal sepsis, binary and multivariate logistic regression analysis were employed. The magnitude of association was measured by using an odds ratio at a 95% confidence interval. Statistical significance was declared at* p*<0.05. Finally, the data were presented in cross tabulations in order to show the distribution of cases and controls.

### 2.6. Ethical Consideration

The ethical approval for the current study was obtained from the University of Health and Allied Sciences Research Ethics Committee before commencing with data collection (UHAS-REC/A.3 [8] 17-18). Approval was then given by the facility management for the study to commence.

## 3. Results

### 3.1. Sociodemographic and Obstetrical Characteristics of Neonates Index Mothers

In the current study, a total of 103 neonates who had sepsis (cases) with their index mothers and 797 neonates who had no sepsis (controls) with their index mothers were enrolled. The majority of mothers were within the age range of 20-29 years, constituting 62 (60.2%) of cases and 460 (57.7%) of controls. Ninety-one (88.3%) cases and 653 (81.9%) controls were married. Christianity was the dominant religion among the mothers of cases and controls, 85.4% and 85.6%, respectively. Fifteen (14.6%) of cases and 96 (12.0%) of controls were not educated. It was also realized that 24 (23.3%) of cases and 106 (13.3%) of controls were housewives by occupation. In relation to the neonates' sociodemographic characteristics, 25 (24.3%) of the cases and 255 (32.0%) controls were found within the age of 4-7 days (Table 1).

### 3.2. Pregnancy and Obstetric History of Neonatal Mothers

Thirty-five (34.0%) of cases and 472 (59.2%) of controls had spontaneous vaginal delivery. It was noted that the majority of the cases 67 (65.0%) were delivered through cesarean section. The majority of mothers, 86 (83.5%) cases and 712 (89.3%) controls, had received antenatal care (ANC) services at least once during pregnancy. The study revealed that the percentage of mothers who had either urinary tract infections or sexually transmitted infections (UTI/STI) during pregnancy was a fraction higher in the cases, 4 (3.9%), than controls, 29 (3.6%). It was also realized that a higher proportion of mothers who had bleeding disorder during the index pregnancy were of controls, 166 (20.8%), than cases, 3 (2.9%). Similarly, the percentage of mothers who had premature rapture of membrane (PROM) was higher in the controls, 116 (14.6%), than cases, 3 (2.9%). This is shown in Table 2.

### 3.3. Maternal Risk Factors of Neonatal Sepsis

Using both bivariate and multivariable logistic regression, only four variables had shown an overall significant effect on risk of neonatal sepsis at the 5% level of significance. Maternal parity was strongly related to the risk of neonatal sepsis (*p*<0.027). It was further noted that primiparous women had 1.89 times higher odds of having neonates with sepsis as compared to multiparous women (COR=1.89; 95% CI (1.050–4.498)). The study found mode of delivery to be statistically associated with neonatal sepsis (*p*<0.001). The study also showed that CS deliveries were the majority among the cases, 67 (65.0%). Bleeding disorder during pregnancy was significantly related to the risk of neonates developing sepsis (*p*<0.001). Further analysis revealed that neonates born to mothers who had bleeding disorders during the index pregnancy were 8.77 times more likely to develop sepsis compared to neonates born to mothers who did not experience bleeding disorder during the index pregnancy (COR=8.769; 95% CI (2.746–28.004)). Premature rupture of membrane (PROM) had significant association with the risk of neonatal sepsis (*p*<0.001) (Table 2).

### 3.4. Neonatal Characteristics

Seventy-four (71.8%) of cases and 662 (83.1%) of controls were delivered between gestational ages of 37-42 weeks. The majority, 80 (77.7%), of the neonates had normal birth weight above 2.5 kg among the cases and the number was 607 (76.2%) for the controls. The proportion of neonates who had an APGAR score <7 at the first minute were higher in the cases, 47 (45.6%), than controls, 231 (29.0%). Similarly, 29 (28.2%) of cases and 112 (14.1%) controls had an APGAR score <7 at the fifth minute. The minority, 14 (13.6%) cases and 126 (15.8%) of controls, were resuscitated at birth. The majority of the neonates had <1-week duration of stay in the facility with 80 (77.7%) of cases and 753 (94.5%) controls (Table 3).

### 3.5. Neonatal Risk Factors of Neonatal Sepsis

Applying both bivariate and multivariate logistic regression analysis, the following showed significant effect on the risk of neonatal sepsis: APGAR scores in the first (*p*<0.001) and fifth (*p*<0.001) minutes, resuscitation at birth (*p*<0.004), duration of stay at the health facility (*p*<0.001), and neonatal age (*p*<0.001). The probability of developing neonatal sepsis increased with increasing neonatal age in both the crude logistic regression analysis and the adjusted one. There was no discernible pattern in the probability of developing neonatal sepsis based on birth weight. In the crude odds analysis, females were less likely (COR=0.79; 95%CI (0.522–1.194)) to develop neonatal sepsis than males. The probability of developing neonatal sepsis was 2.05 times higher among neonates with APGAR scores of <7 in the crude regression model (COR=2.05; CI (1.355–3.120)) than neonates that had an APGAR score >7 in the first minute. This, however, reduced significantly in the second model (AOR=1.42; 95%CI (0.790–2.551)). Similar findings were made for APGAR scores <7 recorded in the fifth minute. In this case the odds of developing neonatal sepsis were 2.39 times higher than neonates that had an APGAR score >7 in the unadjusted model (COR=2.39; 95% CI (1.493–3.849)) (Table 3).

## 4. Discussion

The present study assessed maternal and neonatal risk factors of neonatal sepsis in order to tackle the disease burden and its specific associated problems. The current study finding revealed that the probability that a neonate develops sepsis increased with increasing neonatal age. It was also realized that three-fourths of the cases (78.7%) had early onset of neonatal sepsis (<7days). The present study finding is congruent with the study conducted by Gebremedhin, Berhe, and Gebrekirstos [12] in Ethiopia where they found three-fourths (76.9%) of cases having early onset of neonatal sepsis. Also there was a slightly higher comparable percentage on early onset of neonatal sepsis in other studies conducted in Ethiopia by Gebrehiwot et al. [13] and Woldu et al. [14] which revealed 81.8% and 81.4%, respectively. The early onset of sepsis (EOS) in the present study could be due to ascending infections from the maternal perineum due to bacterial colonization or probably due to direct contact with microorganisms and the newborns body during the delivery process.

The study also revealed that APGAR scores at the first and fifth minutes were significantly (*p*<0.001) associated with the risk of neonatal sepsis. The current finding is consistent with the results of a previous study conducted by Siakwa et al. [12] where they found the APGAR score at the first minute to be strongly associated with the occurrence of neonatal sepsis (*p*≤0.001) in Ghana. Similar findings were also observed in other previous studies in Ethiopia [11, 15].

Resuscitation at birth was found in this study to be statistically (*p*<0.004) associated with the risk of developing neonatal sepsis. Evidence shows that resuscitation of neonates is a risk factor of sepsis among patients with weak immunity, including hospitalized patients, newborns, and the aged [11, 16]. Poor practices and nonadherence to guidelines by health professionals during resuscitation may predispose the neonate with a greater risk of developing sepsis and this might not be different with the current study.

Utomo in 2010 found cesarean section delivery as a variable that was statistically associated with the risk of developing neonatal sepsis, which is consistent with the results of the current study [17]. It is noted that newborns delivered through CS are not exposed to vaginal and fecal bacteria, but they often experience prolonged hospital stay and late initiation of breastfeeding [18, 19]. Late initiation of breastfeeding after CS may deny the neonate the protective effect of colostrum against different pathogenic microbes that have harmful effects to the survival of the newborn baby and its ability to provide immunity for the neonate [20, 21]. The present study findings disagree with Siakwa et al. [11], where they found mode of delivery not to be statistically related with neonatal sepsis (*p*≤0.535).

Furthermore, our study results showed that premature rupture of membrane (PROM) was significantly associated with the risk of neonatal sepsis (*p*<0.001). Several other study findings are consistent with the current study findings [12, 15–25]. Maternal parity was also found in this study to be significantly associated with the risk of the index neonate developing sepsis (*p*<0.027). The current study is consistent Siakwa et al. [11], where they also found parity to be statistically associated with the risk of developing neonatal sepsis (*p*≤0.001). It was further observed that as parity increases their index neonates are less likely to develop sepsis according to the bivariate logistic regression.

Maternal education was observed not to be a significant risk factor of neonatal sepsis. This finding is congruent with Siakwa et al. [11] but inconsistent with earlier findings by Shah et al. [26] where they observed in a case control study in Nepal that maternal education was statistically related with the risk of neonatal sepsis. It is expected that maternal education would enhance mothers' knowledge on how to care for the newborn and also enhance hygienic practices in order to prevent sepsis compared to their noneducated counterparts [27].

The study findings by Siakwa et al. [11] in Ghana and Gebremedhin, Berhe, and Gebrekirstos [12] in Ethiopia did not observe antenatal service utilization as a risk factor of neonatal sepsis. The present study finding is congruent with the above study findings in Ghana and Ethiopia. The current study findings did not observe great difference in neonatal sepsis between women who utilized antenatal care and mothers who did not utilize antenatal care. The majority, 86 (83.5%), of mothers who utilized ANC services had neonates among cases, though antenatal care utilization is vital in lessening the risk factors of adverse birth outcomes including newborn sepsis but that was not the case in the present study.

### 4.1. Limitation of the Study

Since the study was retrospectively done on only admitted neonates born in the hospital, thus the results might lack generalizability to the total population of sepsis cases recorded in the hospital. Lack of proper documentation by different caregivers brought about omission of certain key information of the study and also brought about errors in the identification of cases and controls in the study.

## 5. Conclusion

The study found both maternal and neonatal factors as possible independent risk factors to have a strong association with the risk of neonatal sepsis. The study also observed that the majority of the neonates had early onset of sepsis. Therefore, encouraging mothers to utilize antenatal services might help identify the risk factors and possible interventions to minimize the risk factors of adverse birth outcomes including neonatal sepsis. And also healthcare personnel improving the care they render to mothers and babies could be a key factor in reducing neonatal sepsis.

## Figures and Tables

**Table 1:** Sociodemographic characteristics of cases and controls attending the Trauma and Specialist Hospital.

**Variables**	**Cases**	**Controls**	**Total**
	**n=103 (11.4**%**)**	**n=797 (88.6 **%**)**	**n=900 (100**%**)**
**Maternal age**			
<20	7 (6.8)	49 (6.1)	56 (6.2)
20-29	62 (60.2)	460 (57.7)	522 (58.0)
30-39	33 (32.0)	276 (34.6)	309 (34.3)
40+	1 (1.0)	12 (1.5)	13 (1.4)
**Marital status**			
Never married	12 (11.7)	139 (17.4)	151 (16.8)
Married	91 (88.3)	653 (81.9)	744 (82.7)
Widowed	0 (0.0)	5 (0.6)	5 (0.6)
**Religion**			
Christian	88 (85.4)	682 (85.6)	770 (85.6)
Muslim	15 (14.6)	115 (14.4)	130 (14.4)
**Educational level**			
None	15 (14.6)	96 (12.0)	111 (12.3)
Primary/JHS	50 (48.5)	391 (49.1)	441 (49.0)
SHS	23 (22.3)	158 (19.8)	181 (20.1)
Tertiary	15 (14.6)	152 (19.1)	167 (18.6)
**Occupation**			
Housewife	24 (23.3)	106 (13.3)	130 (14.4)
Civil servant	17 (16.5)	144 (18.1)	161 (17.9)
Trader	53 (51.1)	484 (60.7)	537 (59.7)
Student	9 (8.7)	63 (7.9)	72 (8.0)
**Neonate sex**			
Male	58 (56.3)	402 (50.4)	460 (51.1)
Female	45 (43.7)	395 (49.6)	440 (48.9)
**Neonate age**			
<3	56 (54.4)	255 (32.0)	311 (34.6)
4-7	25 (24.3)	247 (31.0)	272 (30.2)
8-11	14 (13.6)	164 (20.6)	178 (19.8)
12-15	4 (3.9)	78 (9.8)	82 (9.1)
>16	4 (3.9)	78 (9.8)	82 (9.1)

**Table 2:** Bivariate and multivariate logistic regression analysis of maternal risk factors of neonatal sepsis.

**Variables**	**Cases**	**Controls**	**Total**	**Chi-square**	***p* value**	**COR (95**%** CI)**	**AOR (95**%** CI)**
	**n=103 (11.4**%**)**	**n=797 (88.6 **%**)**	**n=900 (100**%**)**				
**Maternal age**				0.517	0.915		
<20	7 (6.8)	49 (6.1)	56 (6.2)			Ref.	Ref.
20-29	62 (60.2)	460 (57.7)	522 (58.0)			1.06 (0.460–2.443)	0.54 (0.165–1.794)
30-39	33 (32.0)	276 (34.6)	309 (34.3)			1.19 (0.50–2.853)	0.59 (0.156–2.240)
40+	1 (1.0)	12 (1.5)	13 (1.4)			1.71 (0.19–2.853)	0.94 (0.072–12.506)
**Educational level**				1.758	0.624		
None	15 (14.6)	96 (12.0)	111 (12.3)			Ref.	Ref.
Primary/JHS	50 (48.5)	391 (49.1)	441 (49.0)			1.22 (0.658–2.268)	0.54 (0.240–1.213)
SHS	23 (22.3)	158 (19.8)	181 (20.1)			1.07 (0.534–2.158)	0.50 (0.201–1.248)
Tertiary	15 (14.6)	152 (19.1)	167 (18.6)			1.58 (0.741–3.385)	0.74 (0.228–2.410)
**Parity**				9.184	**<0.027**		
1	40 (48.6)	448 (56.2)	498 (55.3)			1.898 (1.050–4.498)	0.16 (0.070–0.393)
2	30 (29.1)	177 (22.2)	207 (23.0)			0.890 (0.512–1.547)	0.05 (0.016–0.173)
3+	23 (22.3)	172 (21.6)	195 (21.7)			Ref.	Ref.
**Mode of delivery**				24.823	**<0.001**		
SVD	35 (34.0)	472 (59.2)	507 (56.3)			Ref.	Ref.
Instrumental	1 (1.0)	5 (0.6)	6 (0.7)			0.37 (0.042–3.261)	0.35 (0.036–3.453)
CS	67 (65.0)	316 (39.6)	383 (42.6)			0.35 (0.227–2.539)	0.14 (0.087–0.244)
Vacuum	0 (0.0)	4 (0.5)	4 (0.4)			0.44 (0.382–3.914)	0.23 (0.053–0.319)
**Visited health facility for ANC**				3.096	0.079		
Yes	86 (83.5)	712 (89.3)	798 (88.7)			0.60 (0.343–1.064)	0.46 (0.225–0.960)
No	17 (16.5)	85 (10.7)	102 (11.3)			Ref.	Ref.
**Hypertensive disorder**				0.025	0.875		
Yes	9 (8.7)	66 (8.3)	75 (8.3)			1.0 (0.5126–2.198)	0.596 (0.248–1.433)
No	94 (91.3)	731 (91.7)	825 (91.7)			Ref.	Ref.
**Bleeding disorder**				19.195	**<0.001**		
Yes	3 (2.9)	166 (20.8)	169 (18.8)			8.769 (2.746–28.004)	0.07 (0.020–0.252)
No	100 (97.1)	631 (79.2)	731 (81.2)			Ref.	Ref.
**UTI/STI**				0.15	0.901		
Yes	4 (3.9)	29 (3.6)	33 (3.7)			1.07 (0.368–3.107)	0.85 (0.254–2.899)
No	99 (96.1)	768 (96.4)	867 (96.3)			Ref.	Ref.
**PROM**				10.774	**<0.001**		
Yes	3 (2.9)	116 (14.6)	119 (13.2)			5.677 (0.055–0.565)	0.34 (0.087–1.399)
No	100 (97.1)	681 (85.4)	781 (86.8)			Ref.	Ref.

**Table 3:** Bivariate and multivariate logistic regression analysis on neonatal risk factors of sepsis.

**Variable**	**Cases**	**Controls**	**Total**	**Chi-square**	***p* value**	**COR (95**%**CI)**	**AOR (95**%**CI)**
	**n=103 (11.4**%**)**	**n=797 (88.6 **%**)**	**n=900 (100**%**)**				
**Gestational age (in weeks)**				20.109	<0.056		
<37	25 (24.3)	77 (9.7)	102 (11.3)			Ref.	Ref.
37-42	74 (71.8)	662 (83.1)	736 (81.8)			2.90 (1.742–4.842)	1.70 (0.851–3.408)
>42	4 (3.9)	58 (7.3)	62 (6.9)			4.70 (1.553–14.273)	4.89 (1.367–17.526)
**Birth weight (kg)**				9.571	0.078		
<1.5 kg	11 (10.7)	36 (4.5)	47 (5.2)			Ref.	Ref.
1.51 kg - 2.5 kg	12 (11.7)	154 (19.3)	166 (18.4)			3.92 (1.602–9.597)	2.15 (0.697–6.672)
>2.5 kg	80 (77.7)	607 (76.2)	687 (76.3)			2.31 (1.135–4.736)	0.83 (0.305–2.259)
**Neonate sex**				1.258	0.262		
Male	58 (56.3)	402 (50.4)	460 (51.1)			Ref.	Ref.
Female	45 (43.7)	395 (49.6)	440 (48.9)			0.79 (0.522–1.194)	1.17 (0.718–1.933)
**APGAR score in the first minute**				11.841	**<0.001**		
<7	47 (45.6)	231 (29.0)	278 (30.9)			2.05 (1.355–3.120)	1.42 (0.790–2.551)
≥7	56 (54.4)	566 (71.0)	622 (69.1)			Ref.	Ref.
**APGAR score in the fifth minute**				13.730	<**0.001**		
<7	29 (28.2)	112 (14.1)	141 (15.7)			2.39 (1.493–3.849)	1.89 (0.925–3.896)
≥7	74 (71.8)	685 (85.9)	759 (84.3)			Ref.	Ref.
**Resuscitation at birth**				0.341	<**0.004**		
Yes	14 (13.6)	126 (15.8)	140 (15.6)			Ref.	Ref.
No	89 (86.4)	671 (84.2)	760 (84.4)			0.87 (10.485–1.563)	0.44 (0.177–1.099)
**Crying immediately after birth**				0.215	0.643		
Yes	88 (85.4)	694 (87.1)	782 (86.9)			0.83 (0.462–1.518)	0.92 (0.380–2.264)
No	15 (14.6)	103 (12.9)	118 (13.1)			Ref.	Ref.
**Duration of stay**				70.991	<**0.001**		
<1 week	80 (77.7)	753 (94.5)	833 (92.6)			Ref.	Ref.
2 weeks	8 (7.8)	34 (4.3)	42 (4.7)			0.45 (0.202–1.009)	0.64 (0.243–1.687)
3 weeks	5 (4.9)	7 (0.9)	12 (1.3)			0.14 (0.046–0.479)	0.16 (0.025–1.142)
> 3 weeks	10 (9.7)	3 (0.4)	13 (1.4)			0.03 (0.009–0.118)	0.03 (0.007–0.179)
**Neonate age**				21.417	<**0.001**		
<3	56 (54.4)	255 (32.0)	311 (34.6)			Ref.	Ref.
4-7	25 (24.3)	247 (31.0)	272 (30.2)			2.170 (1.312–3.588)	1.17 (0.647–2.133)
8-11	14 (13.6)	164 (20.6)	178 (19.8)			2.573 (1.387–4.771)	2.50 (1.193–5.237)
12-15	4 (3.9)	78 (9.8)	82 (9.1)			2.910 (1.012–8.371)	2.67 (0.817–8.743)
>16	4 (3.9)	78 (9.8)	82 (9.1)			4.282 (1.505–12.184)	13.28 (3.356–52.589)

## Data Availability

The data used to support the findings of this study are available from the corresponding author upon request.
